# Constant Light Exerted Detrimental Cardiovascular Effects Through Sympathetic Hyperactivity in Normal and Heart Failure Rats

**DOI:** 10.3389/fnins.2020.00248

**Published:** 2020-03-27

**Authors:** Jia-Ni Jing, Zhao-Tang Wu, Miao-Ling Li, Yang-Kai Wang, Xing Tan, Wei-Zhong Wang

**Affiliations:** ^1^Department of Marine Biomedicine and Polar Medicine, Naval Medical Center of People’s Liberation Army (PLA), Naval Medical University, Shanghai, China; ^2^Department of Physiology, Naval Medical University, Shanghai, China; ^3^Key Laboratory of Medical Electrophysiology of Ministry of Education, Institute of Cardiovascular Medical Research, Southwest Medical University, Luzhou, China

**Keywords:** constant light exposure, cardiac function, heart failure, rostral ventrolateral medulla, sympathetic activity

## Abstract

It has been documented that constant light exposure exerts complicated cardiovascular effects. However, a mounting collection of conflicting results did not make it any easier for researchers and physicians to consider the role of light on cardiovascular function. This study was designed to investigate how constant light exposure (24 h light/day) influences the cardiac function in normal and heart-failure (HF) rats. In normal rats, two groups of SD rats were accustomed in 12 h light/12 h dark (LD) or 24 h light (constant light, CL) for 4 weeks. In HF rats which was induced by myocardial infarction (MI) was let recover in LD for 4 weeks. Interestingly, compared with rats in LD environment (ejection fraction, EF%: 93.64 ± 2.02 in LD, 14.62 ± 1.53 in HF-LD), constant light (2 weeks) weakened the cardiac function in normal and HF rats (EF%: 79.42 ± 2.91 in CL, 11.50 ± 1.08 in HF-CL). The levels of renal sympathetic nerve activity and c-fos expression in the rostral ventrolateral medulla (RVLM), a key region controlling sympathetic outflow, were significantly increased in normal and HF rats after constant light (RSNA, Max%: 8.64 ± 0.48 in LD, 20.02 ± 1.24 in CL, 20.10 ± 1.16 in HF-LD, 26.82 ± 1.69 in HF-CL). In conclusion, it is suggested that constant light exposure exerts detrimental cardiovascular effects, which may be associated with the RVLM-related sympathetic hyperactivity.

## Introduction

Environmental stimuli have a universal influence on humans. Environmental light can greatly affect health in a time-, wavelength-, and intensity-related manner (Tapia-Osorio et al., 2013; Alves-Simoes et al., 2016; Barba et al., 2018). Prolonged light exposure is increasingly popular among modern humans (Daugaard et al., 2019). The use of light-emitting electronic devices has dramatically increased recently, leading to a broad influence in health, and safety (Chang et al., 2015). Artificial-light exposure has been shown to alter alertness, melatonin level, and circadian rhythm (Tapia-Osorio et al., 2013; Kelly Glazer Baron, 2014). The relationship between prolonged light exposure and cardiovascular events was well studied (Furlan et al., 2000; Martino et al., 2007; Morris et al., 2016; Reynolds et al., 2017; Singh et al., 2019). Unfortunately, we are still in need of a solid proof concerning the cardiovascular effects of light exposure in normal and heart failure (HF) situations. Constant light (CL) was an extremity of prolonged light exposure, which may occur naturally or artificially. Due to light pollution in public and private areas, constant light is a major part of people’s lives quietly without attracting notice (Huss et al., 2019; Park et al., 2019). Constant light exposure was able to influence people’s behavior and health, making adverse effects on the cardiovascular system reportedly (Furlan et al., 2000; Chang et al., 2015; Reynolds et al., 2017; Benedetto and Contin, 2019; Bohmer et al., 2019). Some papers concerning the same type of light even reported opposite effects (Dauchy et al., 2019; Killgore et al., 2019; Siemiginowska and Iskra-Golec, 2019). A systematic study on constant light is of unquestionable importance for people with cardiovascular illness.

Heart failure (HF) is a severe fatal illness that prevailed in modern society, especially in the elderly population (Conrad et al., 2018). The constant light exposure is not convincingly related to heart failure, while the light is reported to regulate cardiovascular function with the involvements of the circadian rhythm and cardiovascular hormones (e.g., melatonin and cortisol) (Furlan et al., 2000; Thosar et al., 2018; Gordijn, 2019; Hasegawa-Ohira et al., 2019; Lee et al., 2019; Petrowski et al., 2019). Natural sunlight exposure was considered to have a beneficial effect on heart function, while artificial light exposure looked like evil because of the adverse effect (Daugaard et al., 2019; May et al., 2019; Shochat et al., 2019). Considering the complexity of light, the biological effect of light was determined by a couple of factors like wavelength, duration, intensity and so forth. A systemic study of the effect of constant light exposure on cardiac function is crucial for the understanding of this complicated issue.

The sympathetic nerve activity is a hypertensive mechanism that is vital for cardiovascular diseases such as hypertension and heart failure (Dampney, 1994; Guyenet, 2006). It is well known that the rostral ventrolateral medulla (RVLM) is a key area in medulla oblongata for maintaining basal blood pressure (BP) and sympathetic tone (Dampney, 1994; Guyenet, 2006). RVLM is associated with the processing of chronic heart failure (HF) by causing prominent sympatho-excitation (Guyenet, 2006). Interestingly, the modulated light is reported to influence on the autonomic nervous activity in humans, hence changes the cardiac function (Ross et al., 2013). Various lighting conditions including constant darkness, and constant light, could change the norepinephrine (NE) level in organs innervated by sympathetic nerves from different levels, by direct action of the superior cervical sympathetic ganglia and secondary effects of the hormonal alterations (Owman et al., 1982). However, it is not clear if the RVLM plays a role in mediating the cardiovascular effect of light during heart failure. Based on this question, this study will try to solve the following questions: (i) What is the cardiovascular effect of constant light exposure in normal and HF rats; (ii) What is the possible mechanism; (iii) is the RVLM involved in this mechanism.

## Materials and Methods

### Animal Procedures and Protocols

Adult male SD rats (220–270 g) were acquired from Sino−British SIPPR/BK Laboratory Animal Ltd. (Shanghai, China). All procedures were obtained approval of the Institutional Animal Care and Use Committee of Naval Medical University, and all operations in this study were conducted in accordance with the Guide for the Care and Use of Laboratory Animals published by the US National Institutes of Health. All animals were housed in groups of five in transparent acrylic cages (50 cm ^∗^ 30 cm ^∗^ 20 cm) at room temperature (25°C) and had free access to food and water.

Two different lighting conditions were performed in this study. Rats were raised in 12 h light and 12 h darkness (LD), constant light (CL). For LD, lighting was controlled by an automatic electric switch. The light was on from 7 a.m. (Zeitgeber time 0, ZT0) to 7 p.m. (ZT12), and off from 7 p.m. (ZT12) to 7 a.m. (ZT24). In this study, we utilized the white light-emitting diodes, with a wavelength of 465–485 nm, which is near the blue-appearing portion of the visible spectrum. And the light intensity was adjusted to approximately 250–300 lux, which is a commonly used light intensity for animal facilities (Gaston et al., 2013). The settings of lighting conditions were aimed to minimize the biological effect induced by light wavelength and intensity, and made the duration of light be the only object of study. Adding on the lighting conditions, myocardial infarction (MI) procedure (permanent ischemia) was employed. Briefly, the left anterior descending (LAD) artery was ligated. Four weeks later, the MI procedure was verified by echocardiography (EchCG) (Ultrasound diagnostic imaging systems, Esaote, Italy).

**FIGURE 1:**
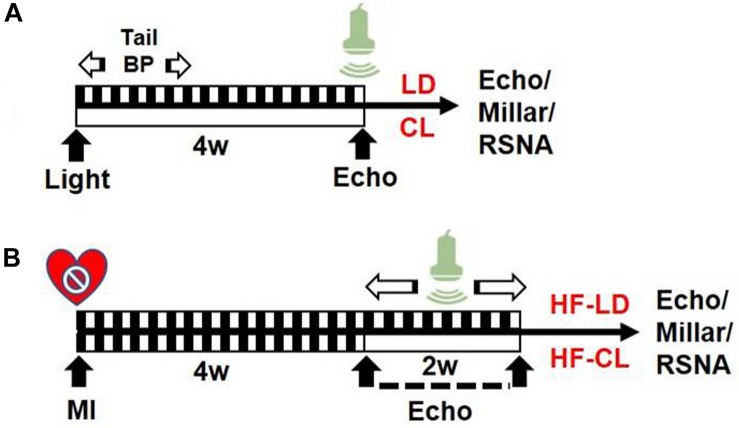
Experiment timeline. **(A)** Schematic design of normal rats in LD/CL. SD rats were accustomed under LD or CL for 4 weeks. In the first week, BP in conscious conditions was measured with tail-cuff system. EchCG was employed in the end for cardiac function. **(B)** Schematic flowchart of HF-LD/HF-CL rats. MI procedure was performed in LD rats and validated by EchCG 4 weeks later. A decrease of EF by 50% was considered HF. Rats were then randomly grouped into two, accustomed under LD or CL for another 2 weeks, in which dynamically monitoring of cardiac function by EchCG was employed in the following 2 weeks. Eventually, rats were sacrificed for intraventricular pressure and RSNA recording.

In this study, rats were grouped as follows: 1. LD or CL rats: normal rats (without MI) in LD/CL for 4 weeks; 2. HF-LD or HF-CL rats: rats were housed in LD for 4 weeks after putting into an MI operation (HF). Cardiac function measurement was performed at the endpoint of each group (see Figure 1).

### Cardiac Function Analysis

Rats were anesthetized with isoflurane (1.5–2.0% in O_2_). EchCG was employed for left ventricular internal systolic diameter (LVIDS), left ventricular internal diastolic diameter (LVIDD), ejection fraction (EF) and fractional shortening (FS), and so forth (Schiller et al., 1989). EF was dynamically monitored for 2 weeks. Basal hemodynamic data were acquired from invasive intraventricular pressure recording with Millar Mikro-Tip pressure catheter. Measured ventricular pressure provides a reliable indication of the contractility or relaxation of the ventricles. In this way, the derivative of pressure over time (dP/dt) and heart rate (HR) were obtained.

### Measurements of BP, HR, and Sympathetic Nerve Activity

As described in our previous work, non-invasive BP measurement using a tail−cuff system (ALC−NIBP, Shanghai Alcott Biotech) was performed to acquire the systolic BP (SBP), diastolic BP (DBP), mean arterial pressure (MAP), and HR in conscious rats (Sun et al., 2018). Rats were accustomed for 15 min before measurement. The results were an average of six repeats. BP and HR were measured daily for the first week after treatments.

Levels of BP, HR, and baseline renal sympathetic nerve activity (RSNA) were also examined in anesthetized state. In anesthetized rats (urethane 800 mg/kg ip and a-chloralose 40 mg/kg ip), the trachea was cannulated and the right femoral artery was catheterized to monitor BP and HR. The left renal sympathetic nerve was isolated retroperitoneally and placed on a pair of silver recording electrodes. RSNA signal was amplified, integrated, and recorded. Data were acquired by a PowerLab system. The maximum of RSNA was measured when the rat was euthanized with an overdose of pentobarbital sodium (200 mg/kg), as reported previously (Wu et al., 2016). Baseline and constant light-exposed RSNA was analyzed as a percentage of maximal RSNA.

### Histology

Immunohistochemistry (IHC) and Immunofluorescence (IF) staining were performed to detect the expression of c-fos in the RVLM (Wu et al., 2016). Briefly, after euthanized with an overdose of pentobarbital sodium (200 mg/kg, ip), rats were perfused transapically with 4% paraformaldehyde (PFA) in 0.1M PBS. Heart and brain were collected and fixed with 4% PFA overnight at 4°C. 20% Sucrose solution was used for dehydration. Frozen sections (20 μm) were incubated with anti-c-fos antibody (1:500, Santa Cruz, sc-8047) followed by sequential development of HRP-DAB or green fluorescent protein (GFP)-conjugated secondary antibody. GFP fluorescence was detected by a laser confocal microscopy (Leica, TCS−SP5). The c-Fos positive cells in the RVLM were determined by DAB-IHC staining, in which the positive cells were dyed brown and counted. The number of total cells in the RVLM were acquired by nuclear staining with hematoxylin. The percentage of c-fos positive neurons in the RVLM equals to brown cells/total cells (Tapia-Osorio et al., 2013).

Masson staining was an optimal means to verify the MI operation, and collagen fibers in the cardiac infarction area after MI operation would be stained in blue. The fibrosis of myocardial infarction area was assessed by Masson staining with computerized planimetry using an image analysis software program., Eosin staining was performed simultaneously as a counterstain.

### Behavioral Observation and Plasma Hormone Examination

In conscious, freely moving rats, spontaneous activity was recorded for three continuous days with DSI implantable telemeters and remote sensing monitoring system (TA-F40PIN 270-0034, DSI, United States). The telemeters were implanted subcutaneously. Rats were let recover for 3 days in LD or CL, respectively. Acclimation of remote sensing monitoring system was carried out 3 times/day for 3 days. Data were analyzed with Dataquest A.R.T system.

Diurnal and nocturnal blood samples were collected. Plasma melatonin, NE and corticosterone were tested with ELISA kit (Shanghai Westang biotech, Inc.). Experiments were conducted carefully following the instruction from merchandise.

### Statistics

Data are expressed as mean ± SEM. Statistical differences between the LD and CL rats were analyzed by Student’s *t-*test. Comparison of MAP and HR acquired by tail-cuff in conscious rats among groups were analyzed by repeated measurement ANOVA followed by Tukey’s *post hoc* test. One−way ANOVA followed by Bonferroni’s *post hoc* test was used for the other multiple comparisons in this study. Differences were considered to be significant by *P* < 0.05.

## Results

### Validation of Heart Failure Model in Rats

**FIGURE 2:**
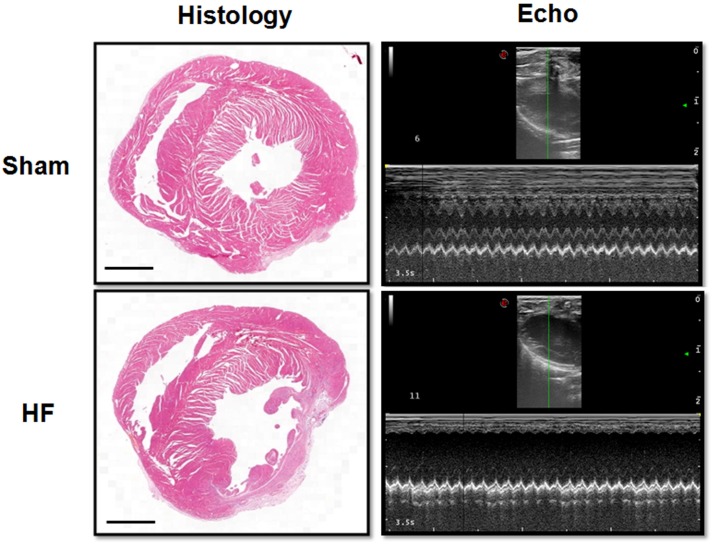
Histology and EchCG evidence after MI. The images from the Histology column showed the eosin staining of the heart from sham and HF rats. Bar = 500 μm. The right two images were representative recordings of the EchCG, from which some of the cardiac function parameters were extracted.

Myocardial infarction (MI) was employed in this study to offer a heart failure (HF) model in SD rats. Briefly, the left anterior descending (LAD) artery was ligated permanently. Four weeks later, the MI procedure was verified by EchCG and intraventricular pressure recording, the cardiac function was significantly reduced after MI (Table 1). From EchCG data, HF group was characterized with higher LVID, and lower FS, EF, and LVPWd, compared with sham. As indicated in Figure 2, histology showed that the LV posterior wall of HF rats was thinner than that of sham rats.

**TABLE 1:** Hemodynamic data of the sham and HF rats recorded with echocardiography and Millar catheter.

	Sham	HF
LVIDd (mm)	5.46 ± 0.15	7.65 ± 0.06^$^
LVIDs (mm)	2.32 ± 0.27	6.05 ± 0.1^$^
HR (min^–1^)	423 ± 20.87	419 ± 18.91
FS (%)	57.56 ± 4.7	20.93 ± 0.93^##^
EF (%)	90.26 ± 3.08	47.86 ± 1.73^¥^
LVPWd (mm)	2.55 ± 0.1	1.86 ± 0.1^##^
+dp/dt (mmHg/s)	5526.54 ± 81.15	3493.39 ± 84.68^##^
−dp/dt (mmHg/s)	−(4926.79 ± 207.8)	−2941.88 ± 227.3^##^
LVESP (mmHg)	152.17 ± 1.75	123.44 ± 6.82^##^
LVEDP (mmHg)	4.88 ± 1.58	20.54 ± 6.31^#^

*Values are mean ± S.E.M. ^#^P < 0.05, ^##^P < 0.01, ^$^P < 0.001, ^¥^P < 0.0001 vs. Sham. n = 5 each group. HF, rats undergone myocardial infarction; LVIDd, LV inner diameter during diastole; LVDIs, LV inner diameter during systole; HR, heart rate; FS, fractional shortening; EF, ejection fraction; LVPWd, LV posterior wall thickness in diastole; + dP/dTmax, maximal first derivative of LV pressure rise; −dP/dTmin, minimal first derivative of LV pressure fall; LVESP, LV end systolic pressure; LVEDP, LV end diastolic pressure.*

### Constant Light Exposure Decreased Cardiac Function in Normal and HF Rats

We performed a one-time detection of cardiac function by EchCG and intraventricular pressure recording at the end of constant light exposure in normal and HF rats. In normal rats, constant light exposure for 4 weeks exerted a mild detrimental effect on cardiac function. Compared with LD, LVIDs was increased, and FS, EF, + dP/dT were decreased in CL rats, implying the myocardial contraction was weakened after constant light exposure. In HF rats, cardiac function was analyzed after LD/CL exposure for 2 weeks (Figure 1B). At the end of constant light exposure, + dP/dT was slightly reduced, yet all the other cardiac function parameters were unchanged. Owing to the severe aggravation of cardiac function after MI, the effect of constant light might be covered.

**FIGURE 3:**
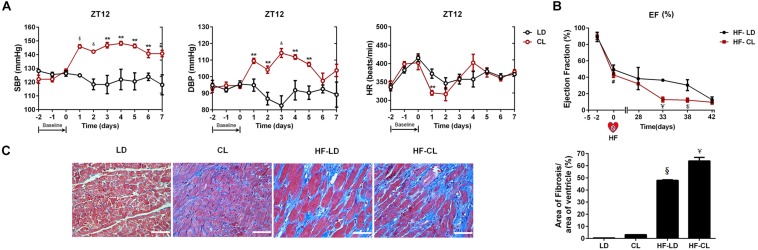
The effect of constant light on cardiac function in normal and HF rats. **(A)** BP measured by tail-cuff system in conscious LD and CL rats. *n* = 6. **(B)** EF acquired by EchoCG dynamical monitoring in HF-LD or HF-CL rats. Day 0 was the day when MI was performed. From Day 0 to day 28, rats were let recover from MI in LD. From day 28 on, rats were housed in LD or CL for 2 weeks in HF-LD or HF-CL rats, respectively. *n* = 5. **(C)** Representative images of left ventricular heart sections stained with Masson staining at ×400 magnification. Scale bars = 100 μm. *n* = 5. Results are performed using one-way ANOVA. *P < 0.05, **P < 0.01, ^&^*P* < 0.001, ^§^ *P* < 0.0001 vs. LD. ^$^*P* < 0.001, ^¥^*P* < 0.0001 vs. HF-LD.

As shown in Figure 3A, BP was measured by tail-cuff system in normal rats, and a rise of BP was observed shortly after constant light exposure. As indicated in Figure 3B, 2-week dynamical monitoring of EF by EchCG was acquired in HF rats since varied light conditions started in CL/LD groups. At day 33 and 38, EF in HF-CL rats was lowered than that in HF-LD rats, which implied that constant light could accelerate the aggravation of cardiac function and later remained stable in HF. At day 42, EF of HF-LD came to the same level with HF-CL, which coincided with the similar level of cardiac function parameters in HF-LD or HF-CL (Table 2). Masson staining (Figure 3C) showed the left ventricular fibrosis in rats. Cardiac fibrosis showed no significant difference between LD and CL rats, and there was an obvious increase in HF-CL rats compared with HF-LD rats.

**TABLE 2:** Hemodynamic data of the LD, CL, HF-LD, and HF-CL rats recorded with echocardiography and Millar catheter.

	LD	CL	HF-LD	HF-CL
LVIDd (mm)	5.46 ± 0.16	5.48 ± 0.38	8.41 ± 0.78**	7.92 ± 0.43^#^
LVIDs (mm)	2.02 ± 0.26	3.12 ± 0.3	7.92 ± 0.67**	7.58 ± 0.43^##^
HR (min^–1^)	433 ± 10.68	397.6 ± 7.1	369.6 ± 25.44	408 ± 14.54
FS (%)	63.24 ± 3.91	43.14 ± 3.03**	5.62 ± 0.65**	4.34 ± 0.42^##^
EF (%)	93.64 ± 2.02	79.42 ± 2.91**	14.62 ± 1.53**	11.50 ± 1.08^##^
LVPWd (mm)	1.81 ± 0.08	1.93 ± 0.26	1.07 ± 0.21	1.28 ± 0.24
+ dp/dt (mmHg/s)	7477.08 ± 604.2	5751.89 ± 69.01**	3211.90 ± 202**	2414.57 ± 138.5^##^
-dp/dt (mmHg/s)	−5952.3 ± 498.8	−5014.1 ± 322.6	−2985.46 ± 122.6**	−2467.51 ± 58.58^##^
LVESP (mmHg)	152.05 ± 5.7	166.02 ± 2.69	145.74 ± 8.96	115.32 ± 5.48^##,$^
LVEDP (mmHg)	0.57 ± 2.68	10.41 ± 2.81	11.58 ± 2.24	10.06 ± 3.58

Values are mean ± S.E.M. **P < 0.01 vs. LD; ^#^P < 0.05, ^##^P < 0.01 vs. CL; ^$^P < 0.05 vs. HF-LD. n = 5 each group. LD, 12/12 light/dark rhythm; CL, constant light; HF-LD, rats undergone myocardial infarction in 12/12 light/dark; HF-CL, rats undergone myocardial infarction in constant light; LVIDd, LV inner diameter during diastole; LVDIs, LV inner diameter during systole; HR, heart rate; FS, fractional shortening; EF, ejection fraction; LVPWd, LV posterior wall thickness in diastole; + dP/dTmax, maximal first derivative of LV pressure rise; -dP/dTmin, minimal first derivative of LV pressure fall; LVESP, LV end systolic pressure; LVEDP, LV end diastolic pressure.

### Sympathetic Activity Was Elevated After Constant Light Exposure in Normal and HF Rats

**FIGURE 4:**
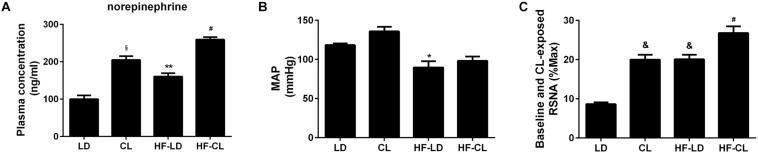
Plasma NE, MAP and baseline RSNA of normal and HF rats. **(A)** Plasma NE concentration (*n* = 5) in four groups; **(B,C)**, MAP and baseline RSNA (%Max) (*n* = 3) statistical diagraphs. *P < 0.05, **P < 0.01, ^&^*P* < 0.001, ^§^ *P* < 0.0001 vs. LD.^ #^*P* < 0.05 vs. HF-LD.

Plasma NE was tested for sympathetic nerve activity (Figure 4A). In normal rats, compared with LD rats, constant light exposure raised plasma NE level. HF increased the NE concentration, and constant light exposure produced the plasma NE to a higher level in HF rats. Furthermore, BP and RSNA were detected in anesthetic rats. BP was significantly reduced after HF developed, while constant light could not change BP in either normal or HF rats (Figure 4B). Baseline RSNA was elevated after constant light exposure in both normal and HF conditions, with the highest level of RSNA for HF-CL (Figure 4C).

### Constant Light Exposure Increased c-fos Expression in the RVLM in Normal and HF Rats

**FIGURE 5:**
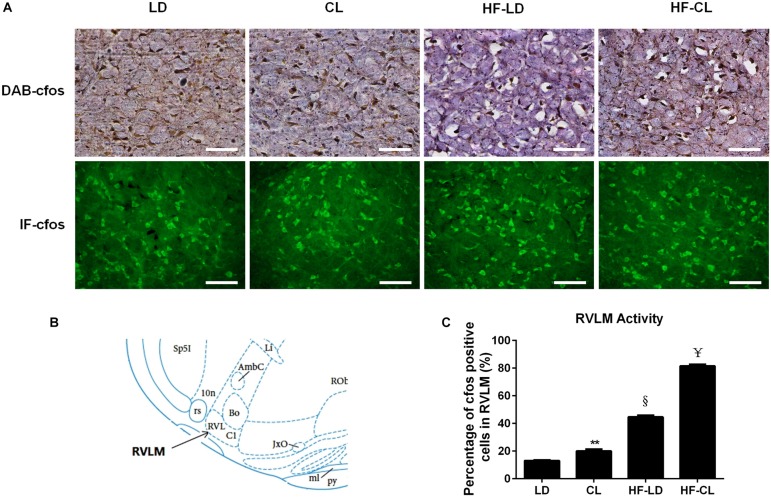
c-fos expression in the RVLM of LD, CL, HF-LD, and HF-CL rats. **(A)** Top: representative IHC-DAB staining of c-fos. Scale bars = 100 μm. Brown showed c-fos expression, and purple for cell nucleus. Bottom: IF-GFP staining of c-fos. Scale bars = 50 μm. Green fluorescence showed c-fos. **(B)** The location of RVLM in brain atlas. **(C)** Percentage of c-fos positive cells in RVLM. Values were extracted from IHC-DAB (**A**, top). **P < 0.01, ^§^ *P* < 0.001 vs. LD. ^¥^*P* < 0.0001 vs. HF-LD. *n* = 5.

IHC-DAB and IF-GFP staining for c-fos were conducted in the RVLM sections collected from LD, CL, HF-LD and HF-CL rats. Constant light exposure raised the expression of c-fos in normal and HF rats (Figure 5). In Figure 5C, the c-fos positive cells in each group were analyzed from the IHC-DAB staining. The percentage of c-fos positive cells increased in CL and HF-LD rats compared with LD group (CL vs. LD: 20.01 ± 0.01% vs. 13.02 ± 0.10%; HF-LD vs. LD: 44.66 ± 0.01% vs. 13.02 ± 0.105). Compared with HF-LD group, the percentage of c-fos positive cells further increased in HF-CL rats (HF-CL vs. HF-LD: 81.53 ± 0.01% vs. 44.66 ± 0.01%).

### Constant Light Exposure Disrupted Circadian Rhythm of Spontaneous Activity, Plasma Melatonin and Corticosterone

**FIGURE 6:**
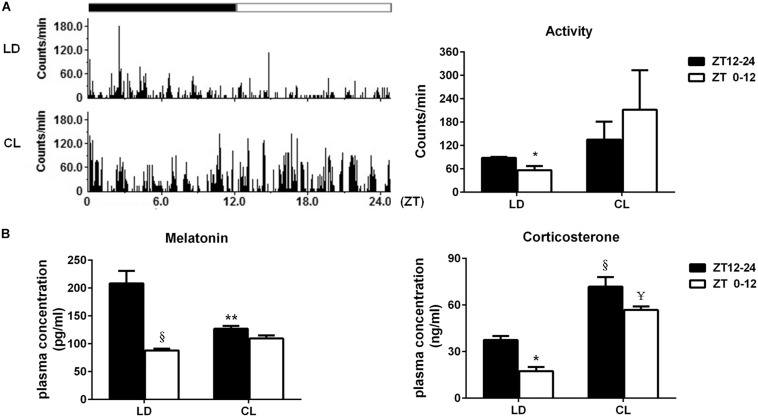
Constant light exposure disrupted circadian rhythm of spontaneous activity, plasma melatonin and corticosterone. **(A)** Left: the spontaneous activity in 24 cycles was recorded by remote sensing monitoring; Right: the average activity difference between ZT12-24 and ZT0-12 in LD and CL groups (*n* = 3). *P < 0.05 vs. ZT12-24 in LD. **(B)** The plasma melatonin and corticosterone level (*n* = 5). Melatonin: **P < 0.01, ^§^ *P* < 0.0001 vs. ZT12-24 in LD. Corticosterone: *P < 0.05, ^§^ *P* < 0.0001 vs. ZT12-24 in LD; ^¥^*P* < 0.0001 vs. ZT0-12 in LD.

The spontaneous activity in conscious, free-moving rats was analyzed in LD and CL rats (Figure 6A). In LD rats, spontaneous activity has a clear circadian rhythm that is the average count in ZT12-24 (dark) was higher than of ZT0-12 (light). Constant light disturbed the circadian rhythm of spontaneous activity. The spontaneous activity count for 24 h indicated that constant light exposure resulted in disappearance of circadian pattern, which normally presented in LD rats. Meanwhile, there was a tendency of increased spontaneous activity in CL group, compared with LD group (*P* > 0.05)., As indicated in Figure 6B, the plasmatic melatonin and corticosterone were decreased in ZT0-12 compared with ZT12-24 under normal lighting conditions, indicating the existence of a circadian rhythm of melatonin and corticosterone. This circadian rhythm in both hormones disappeared after constant light exposure. Moreover, an increase in corticosterone was seen in both ZT12-24 and ZT0-12 in CL rats, showing a possible stress response to constant light in CL rats.

## Discussion

In answering to the questions we raised previously, the effect of constant light on cardiac function was studied in normal and HF rats. First, constant light could deteriorate the cardiac function in normal and HF rats. Second, elevate sympathetic nerve activity was the possible mechanism for this detrimental effect. Finally, sympathetic hyperactivity induced by constant light might be associated with the increased neural activity of RVLM. Circadian rhythm disruption and stress was considered to be involved in the imbalance of RVLM.

Light is a source of energy from the sun or artificial lights. It is a mixture of variant wavelength microwaves. When reviewing the literature about the biological effects of light, the intensity, wavelength, exposure time and duration came into our concern. High intensity of light is a treatment for post-traumatic brain injury patients who are lack of sunlight exposure (Dams-O’Connor et al., 2019). Exposure to red light could result in a time- and dose-dependent thermal hyperalgesia and mechanical allodynia (Khanna et al., 2019). The green light was reported to aggravate high-fat diet-induced obesity and metabolic disorders in male mice (Zhang et al., 2019). Light-at-night (LAN) altered locomotor activities, anxiety and memory for recognition in aged rats in a sex and hormone-dependent manner (Datta et al., 2019). Exposure duration is also a determinant factor of the light biological effect. Insufficient sun exposure causes low plasma Vitamin D, which has an extensive influence across the human body, and eventually causes rickets, immune development, obesity and metabolic disorders in male mice (Bosman et al., 2019; Darling et al., 2019; Memari et al., 2019; Zhang et al., 2019). Greater daytime light exposure by controlled artificial and uncontrolled daytime light is associated with decreased depressive symptoms in bipolar disorder (Esaki et al., 2019). However, prolonged light exposure is also associated with cardiovascular disease and circadian rhythm disorder (Blagonravov et al., 2019). Constant lighting is the extremity of prolonged light exposure. Constant or prolonged light exposure was reported to have a series of actions, such as brain development, psychosocial and emotional disorders, reproductive failure, biliary hyperplasia and liver fibrosis, increased oxidative stress and loss of circadian rhythms in locomotor activity, energy metabolism and insulin sensitivity (Tapia-Osorio et al., 2013; Alves-Simoes et al., 2016; Benedetto and Contin, 2019; Chen et al., 2019; Datta et al., 2019; Fobert et al., 2019; Haraguchi et al., 2019). The biological action of light on cardiovascular system is still unclear.

Constant exposure of white light with moderate intensity was studied in this work. To maximally imitate the regular circumstances, we utilized the LED light with moderate intensity. We employed constant light exposure for 2–4 weeks and checked the effectiveness of the animal model with plasma melatonin and spontaneous activity. According to published literature, duration of constant light exposure differs with a range from 2 to 12 weeks (Alves-Simoes et al., 2016; Abdel Gawad et al., 2019; Saini et al., 2019; Tchekalarova et al., 2019). Before adopting a 2/4-week exposure as our protocol, a bunch of preliminary experiments were performed. In normal rats, BP under conscious conditions was raised shortly after constant light exposure. Spontaneous activity difference between day and night was diminished in 4 weeks of constant light. The diurnal plasma melatonin was not significantly changed after constant light exposure for 4 weeks, while the nocturnal melatonin slumped to the level of its diurnal secretion, making the circadian pattern of melatonin abolished. Moreover, the dynamic monitoring of EF in HF rats indicated that the EF have dropped to 11.50% after constant light exposure in the end of 2 weeks, and the EF of HF-LD (14.62%) and HF-CL were sustained at the same level. This indicated to us the surprisingly powerful action of constant light, which could lower the heart function in fewer than 1 month. However, evidence is insufficient to ascertain this slightly weakening of cardiac function is linked to heart attack from a long-term perspective.

In trying to unveiling the effect of constant light on heart, two different rat models were employed, the normal and HF rats. Heart failure is a progressive heart disease that reduces the pumping action of the myocardium. Coronary artery occlusion is a major cause of heart failure. In this study, MI operation was conducted as a HF challenge. This operation is widely accepted in the study of heart failure and myocardial ischemia/reperfusion. MI followed by reperfusion promotes a complex series of inflammatory reactions as noted in a variety of animal studies, including that in rats (Kain et al., 2014; Prabhu and Frangogiannis, 2016). Since light could impact a broad range of organs, including the immune, endocrine and nervous systems (Hasegawa-Ohira et al., 2019; Memari et al., 2019; Saini et al., 2019). In addition, hormone and automatic nerve can influence the inflammation level remarkably (Chrousos, 2007). Taken together, it became extremely difficult to consider which is the main factor for cardiac function alteration, blood reperfusion or constant light exposure. Permanent ischemic MI model would let us focus on the action of constant light. In addition, we found that constant light merely had a mild to moderate effect in normal rats, yet a relatively greater impact in HF rats. This may attribute from the facts that the sympathetic activity was a central regulator of constant light, and heart failure is also characterized by elevated level of sympathetic activity. Heart failure and constant light-induced sympathetic hyperactivity can create a vicious circle, and thus a synergistic effect on heart function and myocardial fibrosis (Packer, 1990; Kaye et al., 1995). It is possible that heart failure patients may be more susceptible to constant light. This will help to provide a full-scale picture of the effects of constant light, therefore improve the significance of this work.

Cardiac function is a reliable indicator for the severity of heart failure which will provide direct parameters that reflect the ability of ventricular contractility or relaxation. We evaluated the cardiac function in an inclusive manner, including EchCG, invasive intraventricular pressure recording, tail-cuff BP measurement and histology. EchCG and intraventricular pressure recording are crucial for this study. With the combination of non-invasive EchCG and invasive intraventricular pressure recording, we were able to acquire an inclusive data of cardiac function. Compared with LD rats, constant light could lower heart function in normal rats. In the HF rats, constant light did not strongly influence cardiac function, except for the + dP/dT. We further dynamically monitored the EF of HF-LD/HF-CL rats for 2 weeks after MI-induced HF was established. Interestingly, constant light could speed up the exacerbation process of HF with the same endpoint effect. Tail-cuff BP measurement was applied as a complement of cardiac function in normal rats. An increase in BP was first observed after 24 h of constant light exposure, and maintained a high level if constant light continued. Histological evidence of the myocardial infarction was achieved by Masson staining, which showcases the collagen fiber in the surrounding connective tissues in cardiac infarction areas. Constant light increased the fibrosis in HF rats, yet no significant change in normal rats. One limitation in our present study is that certain groups of cardiovascular parameters were not entirely included in every test. However, with the advantage of multiple methods, we considered the incomplete data might not disrupt the consistency and integrity of this paper.

Basal sympathetic tone is essential for cardiovascular homeostasis, and sympathetic hyperactivity is a major cause of hypertension and heart failure (Guyenet, 2006; Johnson and Xue, 2018; Saxena et al., 2018). Light exposure during sleep may enhance the sympathetic nervous system activation (Furlan et al., 2000; Saxena et al., 2018; Hasegawa-Ohira et al., 2019). Sympathetic hyperactivity could enhance heart performance transiently, and we observed an increase in BP shortly after constant light exposure. However, the main cardiovascular effects of constant light exposure might be due to prolonged sympathetic hyperactivity. So we detected the plasma NE and RSNA at the end of constant light exposure for 2/4 weeks. Plasma NE level and RSNA were elevated in normal and HF situations after light exposure, indicating that the constant light exposure increased RSNA persistently. In the condition of prolonged sympathetic hyperactivity, an adverse cardiovascular effect came out since cardiac NE depletion and adrenergic receptor desensitization (Rundqvist et al., 1997). This may explain why constant light exposure played a detrimental effect in normal and HF rats. Moreover, constant light could raise sympathetic activity to a higher level compared with the level in HF-LD. This implied that heart failure and constant light -induced sympathetic hyperactivity can create a vicious circle (Thomas and Marks, 1978; Abraham et al., 1990; Packer, 1990; Kaye et al., 1995; Ramchandra and Barrett, 2015). In summary, elevated sympathetic activity is the bridge to understand the detrimental effect of constant light exposure in both normal and HF rats. RVLM controls the sympathetic tone and regulates the cardiovascular function (Dampney, 1994). We analyzed the neuroactivity of RVLM. Constant light exposure could increase the c-fos expression in RVLM, indicating an elevated sympathetic tone. This coincided with the RSNA data in anesthetic rats.

We further focused on the circadian rhythm (CR). The master pacemaker of CR is located in the Suprachiasmatic Nucleus (SCN) (Hastings et al., 2018). In the SCN, disturbed diurnal rhythm alters gene expression and exacerbates cardiovascular disease (Martino et al., 2007). The circadian rhythm in spontaneous activity, melatonin and corticosterone secretion disappeared, which implied a systemic disturbance of CR after constant light exposure. Collectively, we can infer constant light exposure disrupted the circadian rhythm, which might cause an elevated sympathetic activity by neural and hormonal regulation (Hu et al., 2004; Gamble et al., 2014). Admittedly, we are not able to exclude the influence of stress in constant light model. It was reported that constant light exposure can induce stress and other emotional disorders in rats (Einat et al., 2006; Tapia-Osorio et al., 2013; Bohmer et al., 2019). We also noticed a marked increase in plasma corticosterone and spontaneous activity, which are strong indicators of stress. Stress might also be involved in the sympathetic hyperactivity under constant light (Grippo and Johnson, 2009; Alves-Simoes et al., 2016; Johnson and Xue, 2018).

In the present study, we mainly focused on the role of the RVLM in the hyper-sympathetic effect of constant light. RVLM acts as a dominate regulator of sympathetic nervous activity, while other brain regions may also play an active role. It has been documented that several brain areas such as hypothalamus and medulla oblongata are involved in autonomic processing (Guyenet, 2006). There is a battery of neural nuclei which are active in sympathetic regulation, including paraventricular nucleus (PVN), caudal ventrolateral medulla (CVLM), nucleus of the solitary tract (NTS), RVLM and so forth (Guyenet, 2006). It is well known that the RVLM receives inputs from the PVN, CVLM, and NTS. Although the connection of constant light and these brain nuclei remains to be elucidated, they are considered potential targets for aberrant lighting conditions. Moreover, there is direct evidence that the SCN participates in the light-induced sympatho-excitation (Mutoh et al., 2003). Collectively, in addition to the RVLM, other brain areas (e.g., PVN, CVLM, NTS, and SCN) may also contribute to sympathetic regulation in response to light exposure.

In summary, this study was dedicated to investigating the effect of constant light on cardiovascular activity. We observed a decrease in cardiac function after constant light exposure in both normal and HF rats. The elevated sympathetic activity induced by constant light was fundamental to understand the detrimental effect. RVLM was involved in this action. Circadian rhythm disturbance and stress may be related to sympathetic hyperactivity. This work might shed light on the comprehensive understanding of cardiovascular function in constant light exposure.

## Data Availability Statement

All datasets generated for this study are included in the article/Supplementary Material.

## Ethics Statement

The animal study was reviewed and approved by the Institutional Animal Care and Use Committee of Naval Medical University.

## Author Contributions

J-NJ, Z-TW, and W-ZW: study design. J-NJ, M-LL, and XT: performing experiments. J-NJ, Z-TW, and Y-KW: data collection and analysis. J-NJ and Z-TW: drafting manuscript. Z-TW and W-ZW: revising manuscript content. J-NJ, Z-TW, M-LL, Y-KW, XT, and W-ZW: approving final version of manuscript.

## Conflict of Interest

The authors declare that the research was conducted in the absence of any commercial or financial relationships that could be construed as a potential conflict of interest.

## Supplementary Material

TABLE S1 Raw data for Figures 3–6 and Tables 1, 2.
